# Highly Processed Food Consumption and Its Association with Anthropometric, Sociodemographic, and Behavioral Characteristics in a Nationwide Sample of 2742 Japanese Adults: An Analysis Based on 8-Day Weighed Dietary Records

**DOI:** 10.3390/nu15051295

**Published:** 2023-03-06

**Authors:** Nana Shinozaki, Kentaro Murakami, Shizuko Masayasu, Satoshi Sasaki

**Affiliations:** 1 Department of Social and Preventive Epidemiology, School of Public Health, The University of Tokyo, 7-3-1 Hongo, Bunkyo-ku, Tokyo 113-0033, Japan; 2 Ikurien-naka, 3799-6 Sugaya, Naka-shi 311–0105, Ibaraki, Japan

**Keywords:** highly processed foods, ultra-processed foods, dietary record, Japanese, Japan

## Abstract

This cross-sectional study assessed highly processed food (HPF) consumption and its association with individual characteristics in a nationwide sample of Japanese adults. Eight-day weighed dietary records were obtained from 2742 free-living adults aged 18–79 years across Japan. HPFs were identified based on a classification method developed by researchers at the University of North Carolina at Chapel Hill. The basic characteristics of the participants were assessed using a questionnaire. On average, HPF contributed to 27.9% of daily energy intake. The contribution of HPF to the daily intake of 31 nutrients ranged from 5.7% for vitamin C to 99.8% for alcohol (median, 19.9%). Cereals and starchy foods were the main food groups that contributed to the total energy intake of HPF. Multiple regression analysis showed that the older group (60–79 years) had a lower HPF energy contribution than the younger group (18–39 y) (regression coefficient (β) = −3.55, *p* < 0.0001). Compared to current smokers, past and never-smokers had lower HPF energy contributions (β = −1.41, *p* < 0.02; and −4.20, *p* < 0.0001, respectively). In conclusion, HPFs account for approximately one-third of energy intake in Japan. Younger age and current smoking status should be considered in future intervention strategies to reduce HPF consumption.

## 1. Introduction

Highly processed foods (HPFs), defined as multi-ingredient industrially formulated mixtures [1], are increasingly contributing to diets worldwide [2]. Previous studies have reported that HPF consumption is associated with poor overall diet quality, characterized by increased intake of total fat, saturated fat, trans fat, and free sugars, and decreased intakes of dietary fiber, vitamins (e.g., vitamins A, C, and D), and minerals (e.g., potassium and iron) [2]. Furthermore, high HPF consumption may lead to adverse health outcomes, such as overweight and obesity, cardiovascular and cerebrovascular diseases, metabolic syndromes, depression, and mortality [3,4,5]. Therefore, the official dietary guidelines in several countries recommend reducing HPF consumption [6,7,8,9].

For effective nutrition policies tailored to each population, it is important to identify the factors associated with HPF consumption [10]. Previous studies have evaluated the anthropometric, sociodemographic, and behavioral characteristics associated with HPF consumption and reported several consistent findings. For example, body mass index (BMI) was positively associated with HPF consumption [11,12,13,14,15,16], while age was inversely associated [10,13,14,17,18,19,20,21,22,23,24,25]. However, conflicting results have been found regarding the association between HPF consumption and sex [13,14,17,18,19,20,21,22,24,25], income [13,20,21,22,24], education [10,13,14,17,18,19,20,21,22,24,26], smoking [10,13,17,26], and physical activity [10,17]. Because the human diet is influenced by many other factors, such as culture, vulnerable groups (i.e., those most likely to consume HPFs) may vary between countries [18] and should be investigated on a national or regional basis.

The Japanese diet is considered to be based on dishes and meals made from various unprocessed or less processed foods [27]. However, the literature on HPF consumption and related individual characteristics in Japan is limited. To the best of our knowledge, only two studies have reported HPF consumption among Japanese adults [28,29], of which only one evaluated its association with the characteristics of participants (BMI) [28,29]. These studies were carried out in a single prefecture with small sample sizes [29]. Furthermore, despite the increase in the intake of alcoholic beverages and ready-made foods [30,31], these items were excluded from the previous studies, which may have led to an underestimation of HPF consumption. In addition, the Japanese diet is characterized by some unique features, including meal combinations consisting of a staple food, main dish, and side dish [32], with a high intake of vegetables, greens and beans, refined grains, and sodium [33]. Given the uniqueness of the Japanese diet, the distribution of HPF consumption among the Japanese population and its associated characteristics should be clarified to consider the direction of nutrition policies on HPF.

This cross-sectional study aimed to assess HPF consumption and its association with anthropometric, sociodemographic, and behavioral characteristics in a nationwide sample of Japanese adults.

## 2. Materials and Methods

### 2.1. Study Procedure

This study used data from the Ministry of Health, Labor and Welfare-sponsored Nationwide Study on Dietary Intake Evaluation (MINNADE) survey, designed to elucidate dietary characteristics and eating behaviors across Japan. The details of the survey are provided elsewhere [34]. Briefly, the survey consisted of two rounds of 1-year data collection (first round: from November 2016 to September 2017; second round: from October 2017 to September 2018). The target population was healthy, community-dwelling Japanese people aged 1–79 years in Japan. Initially, 32 of the 47 prefectures, which represent more than 85% of the total population of Japan, were selected to reflect the proportion of the population number in each region of Japan [34,35] based on the geographical diversity and feasibility of the survey. A total of 441 research dietitians consented to participate in the survey and were responsible for recruitment and data collection. Considering the feasibility and human and financial resources, we decided to include 256 individuals (128 males and 128 females) in each of the nine age groups (1–6, 7–13, 14–19, 20–29, 30–39, 40–49, 50–59, 60–69, and 70–79 years) during the first round of data collection (*n* = 2304 in total). Based on the dropout rate in each sex–age category in the first round, 110–119 participants for each sex–age group were recruited in the second round (*n* = 2051 in total). Therefore, the total target number of individuals was 4355. The main inclusion criterion was free-living individuals willing to participate in the survey. Exclusion criteria were dietitians, those living with a dietitian, individuals working with a research dietitian, those who had received dietary counseling from a doctor or dietitian, those undergoing insulin or dialysis treatment, pregnant or lactating women (at the start of the survey), and infants habitually drinking human milk. Night workers were included, but they were asked not to complete a dietary record (DR) on overnight working days and on the days before and after. Participants in this survey were not randomly selected. Only one person per household participated in the study.

### 2.2. Analytic Sample

**Figure 1:**
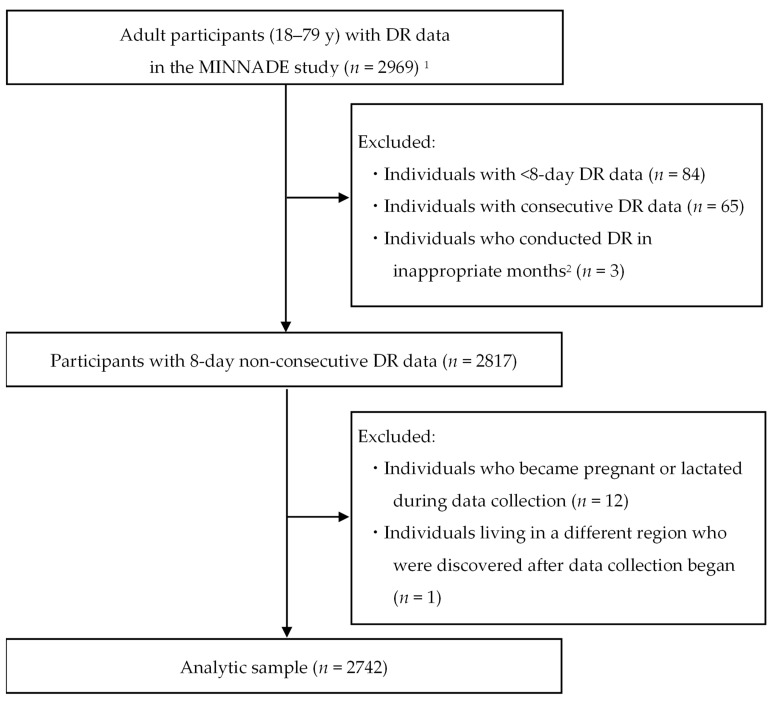
Flow diagram of the selection of participants in the present analysis. DR, dietary record; MINNADE, Ministry of Health, Labour and welfare-sponsored Nationwide study on Dietary intake Evaluation. ^1^ The MINNADE survey aimed to include 4355 individuals and ultimately enrolled 4299 individuals aged 1–79 years. Of these, the number of adults aged 18–79 years with ≥1 day of DR data was 2969. ^2^ Appropriate months were considered October, November, and December for fall; January, February, and March for winter; April, May, and June for spring; and July, August, and September for summer.

A total of 4299 individuals aged 1–79 years participated in the MINNADE survey (first round: *n* = 2263; second round: *n* = 2036). Participants were asked to conduct two non-consecutive days of DRs in each of the four seasons (8 days in total). The number of adults aged 18–79 years with ≥1 day of DR data was 2969 (Figure 1). Among them, we excluded 84 participants with <8-day DR data, 65 participants with at least 2 days of consecutive DR data, and 3 participants who did not perform DRs in the appropriate months (i.e., October, November, and December in fall; January, February, and March in winter; April, May, and June in spring; and July, August, and September in summer). We also excluded 12 participants who lactated or became pregnant during data collection, one who lived in a different geographic area (identified after data collection began), and 62 with missing information on variables of interest. Consequently, the present analysis included 2742 adults aged 18–79 years.

### 2.3. Dietary Assessment

Dietary data were obtained using DRs for a total of 8 days (2 non-consecutive days in each of the four seasons). Details of the DR are provided elsewhere [34]. Briefly, the research dietitians explained verbally and in writing to the participants how to maintain DR. Participants were asked to weigh and record all foods and beverages consumed using a digital scale (KS-812WT; Tanita, Tokyo, Japan) that can measure up to 2 kg in increments of 1 g. The set of two recording days in each season comprised two weekdays (Monday to Friday) for half of the participants and one weekday and one weekend day (Saturday or Sunday, as well as national holidays) for the other half. This approach was used to obtain approximate overall dietary data (3:1 ratio of weekdays to weekends [actually 5:2]) without compromising the feasibility and simplicity of the survey.

The main items recorded were as follows: (i) dish names; (ii) location where the eating occasion occurred (home, restaurants, school or workplace [i.e., food services], or other location); (iii) food names (ingredients included in dishes); and (iv) measured weights or approximate amounts of food consumed. The recording sheets were retrieved by a research dietitian within a few days of each recording day (usually the next day). The research dietitian verified the completeness of the records and recorded additional information if necessary. The research dietitian assigned a food code from the Standard Tables of Food Composition in Japan (STFCJ) [36] to each food item. For packaged foods and home-prepared meals, each ingredient and its weight consumed were estimated as accurately as possible from approximate portion sizes, restaurant and manufacturer websites, ingredient labels, nutritional information on food packages, and cookbooks. Other research dietitians confirmed all food codes and weights in the central office of the study. Daily energy and nutrient intake were calculated for each participant using the STFCJ [36].

### 2.4. Classification of Foods Based on the Degree of Food Processing

A total of 21,936 DRs were obtained from 2742 participants. After excluding dietary supplements, 1,107,350 food items (1989 food codes) were identified. We classified all food items according to their level of processing using the framework developed by researchers at the University of North Carolina (UNC) at Chapel Hill [1]. The UNC system is based on the most widely used food classification system, NOVA [27], but with enhanced definitions of food categories [1,37]. A previous study showed that the UNC system had higher inter-rater reliability than the NOVA classification system [38]. The UNC system classifies food items into four groups: (1) unprocessed and minimally processed; (2) basic processed; (3) moderately processed; and (4) highly processed.

**Figure 2:**
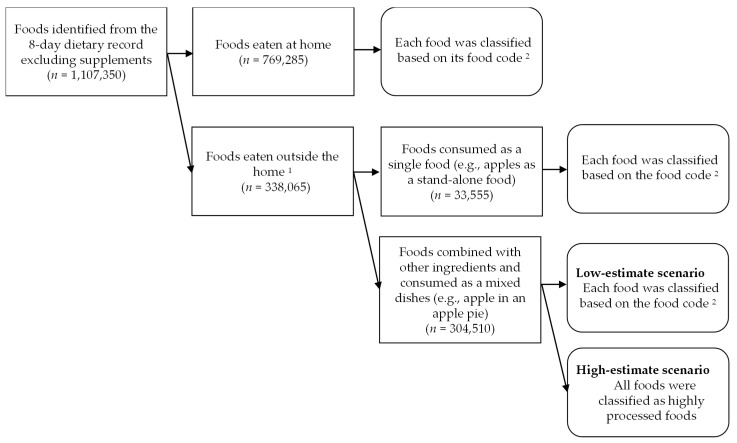
Flow chart of the classification of foods in the 8-day dietary record obtained from 2742 Japanese adults aged 18–69 years. ^1^ Foods eaten in restaurants, cafeterias, or other places outside of the home. ^2^ Food codes from the Standard Tables of Food Composition in Japan (Eighth Revised Edition) were used [36]. Each food was classified into one of the following four categories according to the level of food processing: unprocessed/minimally processed, basic processed, moderately processed, or highly processed, according to the classification system proposed by Poti et al. [1].

Regarding food classification, the Food and Agricultural Organization recommends distinguishing food items processed in industrial settings and those prepared by hand at home or in artisan settings (e.g., street foods) and disaggregating homemade recipes into their ingredients when possible [39]. However, it is difficult to distinguish between artisanal and industrial foods [40], especially for mixed dishes prepared outside the home (e.g., in supermarkets and restaurants), as detailed information on brand names, ingredients, and the preparation process is not always available [26,41]. Therefore, no consensus has been reached on whether to classify food according to the individual ingredients contained or according to the dish as a whole without disaggregating into its ingredients [42]. Therefore, we decided to classify foods consumed outside the home as mixed dishes in two different scenarios: one considers all mixed dishes prepared outside the home as artisanal food and classifies each ingredient based on its food code (low-estimate scenario), while the other considers mixed dishes prepared outside the home as industrial foods and classifies all ingredients into HPFs (high-estimate scenario). A flowchart of food classification is shown in Figure 2. For foods eaten at home (*n* = 769,285), we classified each food ingredient according to its food code. Similarly, foods consumed outside the home as a single item (e.g., apples, black tea, and black coffee) (*n* = 33,555) were classified based on their food code. Meanwhile, foods consumed outside of the home as a mixed dish (*n* = 304,510) were classified according to their food code in the low-estimate scenario and classified into HPFs uniformly in the high-estimate scenario. Taking apple pie as an example, the low-estimate scenario classified individual ingredients, such as apple and butter, based on food codes, while the high-estimate scenario classified all ingredients as HPF.

When foods were classified according to food codes, 421 food codes were identified as HPF. Each food code classified as HPF was further classified into ten food groups based on the STFCJ classification [36] and the similarity of the nutrient composition or culinary use of foods. Examples of HPFs classified according to food codes in each food group are shown in Table S1.

### 2.5. Assessment of Basic Characteristics

Body weight (in 0.1 kg) and height (in 0.1 cm) were measured barefoot and in light clothing by a family member or research dietitian using standardized procedures. Self-reported height and weight were used for participants who could not be measured (*n* = 5). BMI was calculated as body weight (kg) divided by height squared (m^2^).

Information on other characteristics was collected using a questionnaire. The age at the beginning of the study was calculated based on the date of birth. The annual household income was asked in 16 options and reclassified into three categories (<4, ≥4 to <7, and ≥7 million Japanese yen). Similarly, the education level was asked in five categories and reclassified into three categories: junior high school or high school, junior college or technical school, and university or higher. Smoking status was categorized into three categories: current, past, or never. The employment status was categorized as unemployed (including students), part-time, or full-time. For six activities (walking, cycling, standing, running, exercise that causes sweating, and sleeping), self-reported hours spent per day or week during the previous month were asked. Physical activity (as a total metabolic equivalent, h/day) was calculated by summing the product of the self-reported daily hours spent on each activity and the corresponding metabolic equivalent value [43,44,45,46].

### 2.6. Data Analysis

All analyses were performed using the statistical software package SAS version 9.4 (SAS Institute Inc., Cary, NC, USA). Statistical significance was defined as a two-sided *p*-value of <0.05.

First, the basic characteristics of the participants were expressed as mean and standard deviation (SD) for continuous variables and as number and percentage for categorical variables. The mean daily HPF consumption (g) and energy (kJ) was calculated in the low-estimate scenario (all food items were classified according to their food code) and the high-estimate scenario (food items consumed outside the home as a mixed dish were classified as HPF). The difference in estimates between the scenarios was examined using a paired t-test. Pearson’s correlation coefficients were computed to assess the association between HPF consumption and the consumption of foods in other processing categories (i.e., unprocessed/minimally processed, basic processed, and moderately processed foods). The means of HPF intake (g/day and kJ/day) and the weight and energy contribution (%) to total HPF consumption were calculated for each of the 10 food groups. In addition, we calculated the mean daily intake of energy and 31 nutrients from the HPFs and their contribution (%) to the total daily intake. Moreover, differences in HPF weight and energy contributions between categories of basic characteristics (age, sex, BMI, annual household income, educational level, smoking status, employment status, and physical activity) were examined using univariate analysis, followed by Tukey’s test when appropriate. Since parametric and non-parametric tests showed similar results, we show the parametric test results; an unpaired t-test was used for sex, and one-way analysis of variance was used for other variables. Finally, the differences in HPF weight and energy contributions across the categories of basic characteristics were analyzed using multivariable linear regression, with all variables entered into the model simultaneously.

## 3. Results

The basic characteristics of the participants are listed in Table 1. The mean age was 48.4 years (SD, 17.6) and the mean BMI was 23.0 kg/m^2^ (SD, 3.5). The participants were almost equally distributed in the three categories for each annual household income and educational level. Never-smokers and full-time workers represented >60% of the participants. The mean HPF consumption in the low-estimate scenario (447 g/day) was significantly (*p* < 0.0001) lower than in the high-estimate scenario (845 g/day). Similarly, the mean energy intake from HPFs in the low-estimate scenario (2357 kJ/day) was significantly (*p* < 0.0001) lower than in the high-estimate scenario (3547 kJ/day). For both low- and high-estimate scenarios, the weight contribution of HPFs was significantly correlated with that of unprocessed/minimally processed foods (r = −0.41 and −0.66, respectively), basic processed foods (r = −0.45 and −0.60, respectively), and moderately processed foods (r = 0.04 and −0.37, respectively). Similarly, the energy contribution of HPFs in the low- and high-estimate scenarios was significantly correlated with that of unprocessed/minimally processed foods (r = −0.48 and −0.72, respectively), basic processed foods (r = −0.62 and −0.73, respectively), and moderately processed foods (r = −0.11 and −0.49, respectively).

Table 2 shows the HPF consumption of each food group and its percentage contribution to the total HPF consumption. In the low-estimate scenario, the average HPF consumption in grams was the largest for alcoholic beverages among all food groups. In contrast, seasoning and spices (e.g., dashi [stock], soy sauce, and seasoning sauces) had the highest average weight contribution. Meanwhile, in the high-estimate scenario, non-alcoholic beverages were the largest both in weight and weight contribution. Furthermore, regardless of the classification method, cereals and starchy foods had the highest total energy intake from HPFs and contribution to the total energy intake from HPFs.

The energy and nutrient intake from HPFs and their contribution to the total daily intake are shown in Table 3. The mean energy contribution of HPFs in the low-estimate scenario was significantly lower than in the high-estimate scenario (27.9% vs. 42.4%, respectively, *p* < 0.0001). The median HPF contribution to the total daily intake of 31 nutrients was 19.9% in the low-estimate scenario and 35.9% in the high-estimate scenario. In both scenarios, the contribution of HPFs to total daily intake was highest for alcohol (99.8% and 99.9%, respectively), followed by sodium (58.9% and 69.7%, respectively), and lowest for vitamin C (5.7% and 22.8%, respectively).

The percentage contribution of HPFs to the total dietary intake according to the categories of basic characteristics is shown in Table 4. Regardless of the classification method or the unit of contribution (i.e., the percentages of grams or energy), some consistent associations were found between basic characteristics and HPF contribution. For example, the older group (60–79 years) had lower mean weight and energy contributions from HPFs than the younger and middle-aged groups (18–39 and 40–59 years, respectively) (*p* < 0.005). Similarly, females had lower weight and energy contributions from HPFs than males (*p* < 0.005). Moreover, the weight and energy contributions of HPFs were higher in current smokers than in past- or never-smokers, and in full-time workers than in part-time workers or non-workers (*p* < 0.0001 for all).

Table 5 shows the associations between basic characteristics and HPF consumption in the low-estimate scenario. After adjustment for other variables of basic characteristics, the older group (60–79 years) had significantly lower weight and energy contributions of HPFs than the younger group (18–39 years) (*p* < 0.0001). Moreover, compared to current smokers, past and never-smokers had lower weight and energy contributions of HPFs (*p* < 0.02). Furthermore, females had a higher weight contribution of HPF than males (*p* < 0.0001), although no differences were observed between sexes for the energy contribution. All these results were observed similarly in the high-estimate scenario (Table S2). In addition, the results of the high-estimate scenario showed that the middle-aged group (40–59 years) had significantly lower weight and energy contributions than the younger group (18–39 years) (*p* < 0.0001). Moreover, both the weight and energy contributions of HPFs were higher in part-time and full-time workers than in non-workers.

## 4. Discussion

### 4.1. Main Findings

We cross-sectionally examined HPF consumption and its association with anthropometric, sociodemographic, and behavioral characteristics among Japanese adults. The energy contributions of HPFs were 27.9% and 42.4% in the low- and high-estimate scenarios, respectively. In addition, younger age (18–39 years) and current smoking status were associated with higher weight and energy contributions of HPFs. To the best of our knowledge, this is the first study to evaluate HPF consumption and its association with various individual characteristics in a large nationwide sample from a diverse geographic area in Japan.

### 4.2. Scenarios of HPF Consumption

Previous studies have varied in deciding whether to disaggregate mixed dishes prepared outside the home before classifying foods according to the degree of processing [28,47,48]. Disaggregating recipes into ingredients is important to accurately estimate the consumption of culinary ingredients [26]. Meanwhile, this may lead to an underestimation of HPF consumption because some of these ingredients may have been industrially processed [26]. We observed that the average energy contribution of the HPFs in the high-estimate scenario was approximately 1.5 times higher than that in the low-estimate scenario. Similarly, a previous study classified food items on a food frequency questionnaire using upper- or lower-bound scenarios, in which some food items were classified into more and less processed categories, respectively, compared to normal classification [26]. The results showed that in the upper-bound scenario, the energy contribution of the HPFs was nearly double that of the lower-bound scenario. Furthermore, a very recent study using 24 h dietary recall data also observed that the energy contribution of HPFs ranged from 53.4% to 60.1% across the least and most conservative classifications [41]. Therefore, the estimated HPF consumption can differ considerably depending on the assumption of the processing level of each food item. Differences in food classification methods should be noted when comparing results between studies. Meanwhile, the difference in the scenarios did not significantly change most of the observations on the association between HPF consumption.

### 4.3. Contribution of HPFs

The energy contribution of HPFs was 28% or 42%, depending on the scenario. The percentages were comparable to Brazil (24% [17], 25% [16]), South Korea (26%) [18], Japan (30% [29], 38% [28]), Chile (29%) [20], Mexico (30%) [22], France (36%) [13], and Australia (39%) [12,24], but lower than the United Kingdom (51% [15], 53% [25]), Canada (44% [11], 54% [23]), and the United States (59%) [21], and higher than Italy (17%) [19]. As in other countries [15,16,21,26,49], cereals and starchy foods, including bread, contributed the most to the total energy intake of HPFs. Different main food sources have been identified in other studies, such as pre-prepared/ready-to-eat and frozen dishes [11], processed meat [19], and soft drinks [20], probably due to differences in food culture and classification methods for food groups and HPFs. Furthermore, consistent with previous studies [49,50], HPFs greatly contributed to alcohol and sodium intake. One of the main concerns of the Japanese diet is its high sodium intake, which is a factor that reduces the quality of the diet [33]. In these dietary data, seasonings and spices represented 23% (95/421) of all food codes in the HPF and 67% of the sodium intake from the HPF. Therefore, reducing HPF consumption may lead to a decrease in sodium intake, ultimately improving the quality of the Japanese diet.

### 4.4. Participant Characteristics Related to HPF Consumption

HPFs have high palatability, convenience, and affordability, as well as low satiety, all of which may promote overeating and weight gain [11]. Moreover, a recent study found that HPFs have a higher energy intake rate (kcal/min) than unprocessed foods, which may further promote excess energy intake [51]. Previous studies have consistently reported a positive association between HPF consumption and BMI [11,12,13,14,15,16]. However, no association was found between BMI and HPF consumption in the present study. The reasons for this are unclear but can be explained by the relatively narrow distribution of BMI. In addition, the nutritional characteristics of the major HPFs in Japan may differ from those of other countries. Meanwhile, we found an inverse association between age and HPF consumption, consistent with previous studies [10,13,14,17,18,19,20,21,22,23,24,25]. This relationship can be explained by several factors. For example, younger people tend to emphasize the convenience of food [52], which facilitates HPF consumption [53]. Moreover, younger people tend to be more exposed to packaged foods during the formation of eating habits than older generations [17].

In contrast to BMI and age, conflicting results have been reported regarding the association between HPF consumption and sex [13,14,17,18,19,20,21,22,24,25], income [13,20,21,22,24], education [10,13,14,17,18,19,20,21,22,24,26], smoking [10,13,17,26], and physical activity [10,17]. In this study, females had a lower HPF weight contribution than males. This has also been observed for the HPF energy contribution in previous studies [13,14,18,25]. The sex difference may be attributed to differences in eating behavior, food choice, and nutritional strategy between the sexes [54]. 

We did not observe an association between annual household income and HPF consumption, as in a previous study [18]. At the same time, negative associations between annual household income and HPF consumption have been reported in France [13], Australia [24], and the United States [21], and positive associations have been observed in Chile [20] and Mexico [22]. Purchases of HPFs have also been reported to be higher for households with low socioeconomic status in Australia [55] and those with high socioeconomic status in India [56]. Therefore, the relationship between income and HPF consumption may be opposite between high-and middle-income countries (except for Chile). One reason for this may be the cost of the HPFs. For example, the price of processed foods was lower than that of less processed foods in Belgium [57] and the United Kingdom [58] but higher in Brazil [58]. However, evidence of the cost of HPFs versus non-HPFs is not available in Japan. Therefore, further investigations are needed to understand the characteristics of HPF products in Japan.

There is limited evidence on the association between HPF consumption and employment status or physical activity. We observed that HPF consumption was higher in part-time and full-time workers than non-workers, only in the high-estimate scenario. A previous study in Italy reported that retired people were less likely to consume HPF than manual workers [19]. Therefore, non-workers may have lower HPF consumption than workers. This association may be explained by the scarcity of time of workers. In Norway, time scarcity is associated with the consumption of HPF dinner products [14]. Moreover, in Ireland, time pressure has been positively associated with the use of ready-made foods, such as frozen pizza [14]. More research is needed to determine the association between employment status and HPF consumption.

### 4.5. Social Implications

Our findings suggest that the Japanese consume much of their energy and nutrients from HPFs, similar to previous studies in Japan [28,29] and other countries [11,12,13,15,16,17,18,20,21,22,23,24,25]. Given the possible adverse health effects of HPFs [3,4,5], nutrition policies may be necessary to reduce HPF consumption. According to our results, reducing HPF may decrease sodium intake and, consequently, improve the quality of the diet. In fact, previous studies have reported that higher HPF consumption was associated with unfavorable profiles of nutrient intake among Japanese adults [28,29]. Our findings also suggest that young people and smokers may benefit from public policies and programs to reduce HPF intake. Nutrition education to reduce HPF consumption may be especially important for young people, as they are likely to maintain a lifelong diet in the future [10]. Other personal characteristics not examined in this study, such as food literacy and beliefs, require further investigation.

### 4.6. Strengths and Limitations

The strength of this study is the use of 8-day DRs collected throughout the year from a large sample of males and females of a wide range of ages from different regions of Japan. This allowed us to represent Japanese dietary habits by considering day-to-day and seasonal variations [59]. However, this study has several limitations. First, although the sampling was carried out to reflect the percentage of population in each region, the study population was not a nationally representative sample of the general Japanese population, but volunteers. Given the burden of data collection, participants may be more attentive to their diets and have healthier eating patterns than the general population. Compared with the nationally representative sample, the distributions of household income, body height, weight, and BMI in this population were similar, although the level of education was somewhat higher [34]. Therefore, there is no strong evidence that the participants in this study differed significantly from the general Japanese population. Second, the UNC system was developed for food supply in the United States and may not be the most appropriate for classifying foods sold in Japan. However, the UNC system is based on the NOVA classification, the most widely used system in many countries, including Japan [28,29]. Furthermore, the UNC system provides detailed definitions and examples of foods in each processing category and has been applied in several countries other than the United States [60,61]. Third, the classification of foods by food codes was performed by a single author and may contain some misclassifications, although the UNC system has high inter-rater reliability [38]. Fourth, although physical activity was estimated according to the methods used in previous studies [45,46], the validity of the estimates has not been investigated. Therefore, the association between physical activity and HPF consumption should be examined more rigorously in future studies. Fifth, other participant characteristic variables were not available in this study and their association with HPF consumption was unknown. Other drivers of HPF consumption include low sociability, adverse life events, screen time, time scarcity, and poor sleep quality [14,19,62,63]. More research should be carried out to identify these factors. Sixth, owing to the cross-sectional nature of the present analysis, more research is warranted to determine the causal relationship between the degree of food processing and the anthropometric measurements. Finally, self-reported dietary data are affected by, for example, reactivity and social desirability bias [64], which may lead to a lower intake of unhealthy foods and underestimate the contribution of HPF.

## 5. Conclusions

Our findings suggest that HPFs account for >25% of energy intake in Japanese adults. Younger age and current smoking may be risk factors for high HPF consumption and should be considered in future intervention strategies to reduce HPF consumption. Since HPF greatly contributes to sodium intake, reducing HPF may improve the quality of diet of the Japanese population. Moreover, given the differences between the socioeconomic and behavioral subgroups, more research is needed to identify the factors that trigger the purchase and consumption of HPFs and develop different approaches for vulnerable groups.

## Figures and Tables

**Table 1:** Basic characteristics of the study participants (*n =* 2742).

Variable	Values ^1^		
Age (y)	48.4	±	17.6
Female (*n*, %)	1392		(50.8)
Body height (cm)	162.7	±	9.0
Body weight (kg)	61.1	±	12.1
Body mass index (kg/m^2^)	23.0	±	3.5
Annual household income, *n* (%)			
<4 million Japanese yen	970		(35.4)
≥4 to <7 million Japanese yen	926		(33.8)
≥7 million Japanese yen	846		(30.9)
Educational level, *n* (%)			
Junior high school or high school	1092		(39.8)
Junior college or technical school	819		(29.9)
University or higher	831		(30.3)
Employment status, *n* (%)			
Unemployed (including students)	622		(22.7)
Part-time job	336		(12.3)
Full-time job	1784		(65.1)
Smoking status, *n* (%)			
Current smoker	452		(16.5)
Past smoker	607		(22.1)
Never smoker	1683		(61.4)
Physical activity level (MET × h)	39.2	±	5.9
Energy intake (kJ)	8391	±	1931
Consumption of foods classified by level of food processing (g/day): low-estimate scenario			
Unprocessed or minimally processed food	1080	±	435
Basic processed food	1066	±	400
Moderately processed food	131	±	68
Highly processed food	447	±	303
Consumption of foods classified by level of food processing (g/day): high-estimate scenario			
Unprocessed or minimally processed food	895	±	421
Basic processed food	887	±	363
Moderately processed food	96	±	62
Highly processed food	845	±	493
Energy intake from foods classified by level of food processing (kJ/day): low-estimate scenario			
Unprocessed or minimally processed food	2155	±	795
Basic processed food	3096	±	997
Moderately processed food	783	±	368
Highly processed food	2357	±	1048
Energy intake from foods classified by level of food processing (kJ/day): high-estimate scenario			
Unprocessed or minimally processed food	1742	±	843
Basic processed food	2526	±	983
Moderately processed food	575	±	346
Highly processed food	3547	±	1522

MET, metabolic equivalent. ^1^ Values are expressed as mean ± standard deviation, unless otherwise indicated. Some percentages do not total 100 because of rounding.

**Table 2:** Mean daily intake (g/day and kJ/day) of highly processed foods from each food group and their weight and energy contribution (%) ^1^ to the total intake of highly processed foods in 2742 Japanese adults.

	Low-Estimate Scenario ^2^								High-Estimate Scenario ^2^							
	g/day		% of Grams		kJ/day		% of Energy		g/day		% of Grams		kJ/day		% of Energy	
Food Group	Mean	SD	Mean	SD	Mean	SD	Mean	SD	Mean	SD	Mean	SD	Mean	SD	Mean	SD
Cereals and starchy foods	62.2	46.1	17.0	12.7	622	102	26.6	14.8	145.9	92.5	19.4	10.8	1151	159	32.2	12.8
Fruits, vegetables, and pulses	7.7	26.5	2.0	6.6	25	18	1.2	3.7	70.2	69.5	8.1	7.5	156	37	4.3	4.3
Meat, fish, and eggs	26.7	21.4	7.6	6.9	280	63	12.3	10.4	66.6	46.5	8.6	5.5	603	110	16.4	9.4
Dairy products	15.2	27.5	3.9	6.0	114	32	4.9	5.5	22.5	34.6	3.0	4.2	140	36	4.1	4.2
Confectioneries	33.1	29.1	9.4	8.9	455	96	19.2	14.4	33.2	29.1	5.4	6.1	456	96	13.8	11.6
Alcoholic beverages	127.5	253.4	17.6	24.9	340	155	11.6	17.8	127.5	253.4	11.9	18.9	340	155	8.5	14.2
Non-alcoholic beverages	66.5	118.3	12.7	16.8	100	43	4.0	6.7	262.6	289.2	26.1	19.0	114	44	3.0	4.4
Fats and oils	8.8	6.4	2.6	2.5	189	34	8.5	6.0	11.9	7.4	1.8	1.6	306	46	8.8	4.8
Seasonings and spices	99.0	63.7	27.2	17.1	232	23	11.7	7.3	103.3	64.4	15.6	11.9	279	27	8.9	4.7
Pickles	0.2	0.8	0.1	0.3	1	1	0.1	0.3	1.1	2.1	0.1	0.3	4	2	0.1	0.2

SD, standard deviation. ^1^ Weight contribution (%) = total weight of highly processed foods consumed in each food group (g)/total weight of highly processed foods consumed (g) × 100. Energy contribution (%) = total energy intake from highly processed foods in each food group (kJ)/total energy intake from highly processed foods (kJ) × 100. ^2^ Among foods eaten outside the home, those combined with other ingredients and consumed as mixed dishes were individually classified according to the food code in the low-estimate scenario. Meanwhile, the high-estimate scenario classified all such foods as highly processed foods.

**Table 3:** Energy and nutrient intake from highly processed foods and their contribution (%) to total daily intake in 2742 Japanese adults.

	Low-Estimate Scenario ^1^				High-Estimate Scenario ^1^			
	Intake		Contribution (%)		Intake		Contribution (%)	
Variable	Mean	SD	Mean	SD	Mean	SD	Mean	SD
Energy (kJ/day)	2357	1048	27.9	9.5	3547	1522	42.4	15.0
Protein (g/day)	15.0	6.1	20.7	7.9	26.8	12.3	37.2	16.4
Total fat (g/day)	19.5	9.7	29.4	11.5	29.8	14.9	45.1	17.9
Saturated fatty acid (g/day)	6.25	3.41	32.5	13.3	8.89	4.65	46.7	18.1
Monounsaturated fatty acid (g/day)	7.50	4.00	29.7	12.2	11.55	6.19	45.8	18.7
Polyunsaturated fatty acid (g/day)	3.74	1.97	28.6	11.9	5.83	2.97	45.0	18.9
n-3 polyunsaturated fatty acid (g/day)	0.42	0.29	22.0	14.3	0.76	0.51	39.4	23.2
n-6 polyunsaturated fatty acid (g/day)	3.14	1.65	29.8	12.1	4.83	2.45	46.2	18.6
Cholesterol (mg/day)	45	30	14.5	9.6	110	74	34.7	20.9
Carbohydrate (g/day)	65.6	29.2	25.3	9.6	101.9	44.9	39.4	15.1
Total dietary fiber (g/day)	2.8	1.5	14.6	8.2	6.1	3.1	31.9	17.0
Sodium (mg/day)	2361	708	58.9	9.5	2778	800	69.7	12.0
Potassium (mg/day)	392	161	16.2	7.4	769	387	32.0	16.9
Calcium (mg/day)	109	58	22.9	11.9	168	82	35.9	18.1
Magnesium (mg/day)	51	20	20.0	7.6	89	40	35.2	16.1
Phosphorus (mg/day)	225	87	21.9	8.0	375	163	36.9	16.0
Iron (mg/day)	1.5	0.6	19.7	7.5	2.7	1.3	36.2	16.9
Zinc (mg/day)	1.3	0.5	15.0	6.1	2.7	1.4	31.9	15.9
Copper (mg/day)	0.16	0.07	14.3	6.7	0.33	0.16	30.2	15.6
Manganese (mg/day)	0.49	0.20	13.9	6.1	1.03	0.56	28.8	14.2
Vitamin A ^2^ (µg/day)	37	25	8.0	6.2	149	172	29.5	21.6
Vitamin D (µg/day)	0.5	0.5	9.3	10.4	1.7	1.7	29.4	24.7
α-Tocopherol (mg/day)	2.2	1.3	28.0	13.4	3.4	1.8	44.3	19.8
Vitamin K (µg/day)	14.3	8.9	7.3	5.8	53.3	42.6	26.0	20.8
Thiamin (mg/day)	0.22	0.13	20.9	11.1	0.37	0.20	36.2	18.4
Riboflavin (mg/day)	0.25	0.12	19.9	9.9	0.42	0.20	34.4	16.9
Niacin (mg/day)	3.5	2.0	19.2	9.4	6.5	3.7	36.2	18.4
Vitamin B6 (mg/day)	0.19	0.12	14.7	8.1	0.40	0.24	31.2	17.8
Vitamin B12 (µg/day)	0.6	0.5	12.2	8.9	1.7	1.5	31.2	22.6
Folate (µg/day)	41	19	13.1	7.3	93	52	29.6	17.3
Vitamin C (mg/day)	5	7	5.7	6.9	21	18	22.8	18.7
Alcohol (g) ^3^	9.3	18.5	99.8	1.8	9.3	18.5	99.9	1.6

SD, standard deviation. ^1^ Among foods eaten outside the home, those combined with other ingredients and consumed as mixed dishes were individually classified according to the food code in the low-estimate scenario. Meanwhile, the high-estimate scenario classified all such foods as highly processed foods.^2^ Retinol equivalent. ^3^ One participant with zero alcohol intake was not included in the calculation of the contribution of alcohol intake.

**Table 4:** Percentage contribution of highly processed foods (in weight and energy) estimated from 8-day dietary records according to categories of basic characteristics among 2742 Japanese adults.

		Low-Estimate Scenario ^1^								High-Estimate Scenario ^1^							
		% of Grams				% of Energy				% of Grams				% of Energy			
Variable	n	Mean		SD	*p* ^2^	Mean		SD	*p* ^2^	Mean		SD	*p* ^2^	Mean		SD	*p* ^2^
Age group (y)					<0.0001				0.005				<0.0001				<0.0001
18–39	972	17.4	^a^	9.5		29.0	^a^	10.2		36.5	^a^	14.0		49.2	^a^	14.3	
40–59	892	17.3	^a^	9.9		28.9	^a^	9.6		34.6	^b^	14.2		45.0	^b^	13.3	
60–79	878	14.0	^b^	7.1		25.5	^b^	8.2		21.1	^c^	11.5		32.1	^c^	11.7	
Sex					<0.0001				0.005				<0.0001				<0.0001
Male	1350	18.8		10.1		28.4		10.3		33.9		15.3		43.8		15.7	
Female	1392	13.9		7.2		27.4		8.7		28.1		14.0		41.0		14.3	
Body mass index (kg/m^2^) ^3^					0.10				0.64				0.51				0.71
T1 (median: 19.8)	914	16.1		9.1		27.9		9.8		31.1		14.9		42.6		14.8	
T2 (median: 22.5)	914	16.2		9.0		27.7		9.5		30.8		14.9		42.0		14.9	
T3 (median: 26.1)	914	16.6		9.2		28.0		9.3		30.9		15.1		42.5		15.4	
Annual household income (Japanese yen)					0.003				0.40				<0.0001				<0.0001
<4 million	970	15.5	^a^	8.6		27.6		9.6		27.8	^a^	14.8		40.1	^a^	15.9	
≥4 to <7 million	926	16.6	^b^	9.1		28.2		9.5		31.9	^b^	15.3		43.2	^b^	15.0	
≥7 million	846	16.9	^b^	9.6		27.8		9.5		33.4	^b^	14.0		44.1	^b^	13.7	
Educational level					0.002				0.07				<0.0001				<0.0001
Junior high school or high school	1092	16.0	^a^	9.2		27.4		9.4		27.3	^a^	14.6		38.8	^a^	14.7	
Junior college or technical school	819	15.7	^a^	8.7		28.1		9.5		32.5	^b^	15.0		44.5	^b^	15.1	
University or higher	831	17.2	^b^	9.3		28.3		9.7		34.2	^b^	14.3		45.0	^b^	14.5	
Employment status					<0.0001				<0.0001				<0.0001				<0.0001
unemployed (including students)	622	13.9	^a^	7.0		25.7	^a^	8.4		19.5	^a^	10.6		32.1	^a^	12.6	
Part-time job	336	14.9	^a^	7.4		27.3	^b^	8.1		24.0	^b^	12.0		36.2	^b^	12.6	
Full-time job	1784	17.4	^b^	9.8		28.7	^c^	10.0		36.2	^c^	14.0		47.1	^c^	14.0	
Smoking status					<0.0001				<0.0001				<0.0001				<0.0001
Current smoker	452	21.1	^a^	11.5		31.1	^a^	11.4		38.3	^a^	16.2		48.7	^a^	15.9	
Past smoker	607	17.9	^b^	9.1		28.8	^b^	9.1		31.4	^b^	14.3		41.9	^b^	14.1	
Never smoker	1683	14.4	^c^	7.7		26.6	^c^	8.8		28.8	^c^	14.2		40.9	^b^	14.7	
Physical activity level (MET × h) ^3^					0.04				0.15				0.03				0.003
T1 (median: 33.3)	914	15.9	^a^	9.0		27.6		9.1		31.2	^ab^	14.9		42.4	^ab^	14.6	
T2 (median: 38.3)	914	16.0	^a^	8.9		27.7		9.6		29.9	^a^	14.7		41.2	^a^	15.0	
T3 (median: 45.0)	914	16.9	^a^	9.4		28.4		9.8		31.7	^b^	15.2		43.5	^b^	15.4	

MET, metabolic equivalent; SD, standard deviation; T, tertile. ^a,b,c^ Different superscript letters indicate significant differences between categories (Tukey’s test). ^1^ Among foods eaten outside the home, those combined with other ingredients and consumed as mixed dishes were individually classified based on the food code in the low-estimate scenario. Meanwhile, the high-estimate scenario classified all such foods as highly processed foods. ^2^ Unpaired *t*-test was used for sex, and one-way ANOVA was used for other variables. ^3^ The subjects were categorized into tertiles.

**Table 5:** Associations between basic characteristics and consumption of highly processed foods when foods classified using the low-estimate scenario ^1^ in the 8-day dietary record of 2742 Japanese adults.

	% of Grams				% of Energy			
Variable	Regression Coefficient	95% Confidence Interval		*p* ^2^	Regression Coefficient	95% Confidence Interval		*p* ^2^
Age group (y)								
18–39	Ref	-	-	-	Ref	-	-	-
40–59	−0.58	−1.38	0.22	0.16	−0.45	−1.32	0.43	0.32
60–79	−3.19	−4.11	−2.27	<0.0001	−3.55	−4.56	−2.55	<0.0001
Sex								
Male	Ref	-	-	-	Ref	-	-	-
Female	−3.47	−4.20	−2.73	<0.0001	0.41	−0.40	1.21	0.32
Body mass index (kg/m^2^) ^3^								
T1 (median: 19.8)	Ref	-	-	-	Ref	-	-	-
T2 (median: 22.5)	0.74	−0.60	2.08	0.28	0.73	−0.74	2.20	0.33
T3 (median: 26.1)	0.12	−1.34	1.58	0.87	0.91	−0.69	2.51	0.26
Annual household income ^1^								
<4 million Japanese yen	Ref	-	-	-	Ref	-	-	-
≥4 to <7 million Japanese yen	0.10	−0.68	0.87	0.81	−0.25	−1.10	0.60	0.57
≥7 million Japanese yen	0.42	−0.41	1.24	0.32	−0.70	−1.60	0.20	0.13
Educational level								
Junior high school or high school	Ref	-	-	-	Ref	-	-	-
Junior college or technical school	−0.50	−1.31	0.31	0.23	0.06	−0.83	0.95	0.89
University or higher	−0.28	−1.10	0.54	0.50	0.30	−0.59	1.20	0.51
Employment status								
Unemployed (including students)	Ref	-	-	-	Ref	-	-	-
Part-time job	0.88	−0.27	2.02	0.13	0.99	−0.27	2.24	0.12
Full-time job	0.83	−0.12	1.78	0.09	0.56	−0.48	1.61	0.29
Smoking status								
Current smoker	Ref	-	-	-	Ref	-	-	-
Past smoker	−2.37	−3.42	−1.32	<0.0001	−1.41	−2.56	−0.25	0.02
Never smoker	−4.82	−5.76	−3.87	<0.0001	−4.20	−5.23	−3.17	<0.0001
Physical activity level (MET × h) ^3^								
T1 (median: 33.3)	Ref	-	-	-	Ref	-	-	-
T2 (median: 38.3)	0.36	−0.42	1.14	0.37	0.13	−0.73	0.98	0.77
T3 (median: 45.0)	0.79	0.00	1.58	0.05	0.45	−0.41	1.32	0.31

MET, metabolic equivalent; SD, standard deviation; T, tertile. ^1^ Among foods eaten outside the home, those combined with other ingredients and consumed as mixed dishes were individually classified based on the food code. ^2^ Multivariable linear regression was used including all variables in the model simultaneously. ^3^ The subjects were categorized into tertiles.

## Data Availability

The datasets generated and analyzed during the present study are not publicly available due to restrictions imposed by the Ministry of Health, Labour and Welfare.

## Supplementary Materials

The following supporting information can be downloaded at: https://www.mdpi.com/article/10.3390/nu15051295/s1, Table S1. Examples of highly processed foods classified based on the food code in each food group; Table S2. Associations of highly processed food consumption when foods consumed as a dish consisting of multiple ingredients were classified as highly processed food (high-estimate scenario) with basic characteristics of Japanese adults (*n =* 2742).
