# Supplementary material for: Highly Processed Food Consumption and Its Association with Anthropometric, Sociodemographic, and Behavioral Characteristics in a Nationwide Sample of 2742 Japanese Adults: An Analysis Based on 8-Day Weighed Dietary Records
Source: Nutrients. 2023 Mar 6;15(5):1295. doi: 10.3390/nu15051295 (PMC10005625; doi:10.3390/nu15051295)
Supplement: Supplementary file 1 [file nutrients-15-01295-s001.zip › nutrients-2220844-supplementary.pdf]

Table S1. Examples of highly processed foods classified according to the food code in each food group[1]

**Food Group**	**Examples (Food codes in brackets are based on the Standard Tables of Food Composition in Japan 2020 –Eighth revision–)**
Cereals and starchy foods	[1024] Premixed flour for pancake; [1025] premixed flour for Tempura; [1026] white bread; [1028] white long roll bread; [1031] French bread; [1032] rye and wheat bread; [1034] soft roll bread; [1035] croissants; [1036] English muffins; [1056] instant Chinese noodles, dried by frying, seasoned; [1057] instant Chinese noodles, dried by frying, not seasoned; [1058] instant Chinese noodles, dried without frying; [1060] "yakisoba", instant Chinese oil-fried noodles, dried by frying and in cups; [1061] instant Chinese noodles, dried by without frying and packed in cups; [1062] instant "udon" noodles dried by frying and packed in cups; [1071] wheat gluten, powdered type; [1074] outer steamed wheat "Jiaozi "(Chinese meat dumpling) dough ; [1075] outer steamed wheat "Shumai" (Chinese meat dumpling) dough ; [1076] pizza crust; [1077] bread crumbs, fresh; [1079] bread crumbs, dry ; [1118] rice, glutinous rice products, "Sekihan" (steamed rice with adzuki beans or cowpeas); [1137] corn, cornflakes; [1146] premixed flour for "Okonomiyaki" (Japanese-style savory pancake with various ingredients); [1147] premixed flour for fried food; [1171] premixed flour for Tempura; [11191] soy-sauce flavored instant Chinese noodles, dried by frying and packed in cups
Fruits, vegetables, and pulses	[4041] Soybeans, tofu, "Ganmodoki" (fried mixture of crushed tofu, vegetables and ground yam); [4053] soybeans, soy milk, reconstituted; [4054] soybeans, soy milk based beverage, coffee flavored
Meat, fish, and eggs	[10376] "Kanifumi-kamaboko" (imitation crab meat made from surimi); [10379] "Mushi-kamaboko" (steamed kamaboko); [10381] "Yaki-chikuwa" (baked tubular kamaboko); [10383] "Tsumire" (boiled red meat fish paste); [10384] "Naruto" (boiled stick kamaboko with cross section of red swirl); [10385] "Hanpen" (boiled fishcake made of surimi, yam paste and starch); [10386] "Satsuma-age" (fried surimi); [10388] fish sausage; [10390] breaded and friedhorse mackerel with integument; [10416] crustacean, whiteleg shrimp, tempura; [11178] pork, ham, pressed; [11186] pork sausage, Vienna; [11189] pork sausage, Frankfurter; [11190] pork sausage, Bologna; [11276] pork, large type breed, loin, "Tonkatsu"(breaded and fried pork, lean and fat); [11279] pork, large type breed, fillet, lean, "Tonkatsu"(breaded and fried pork); [11289] "Karaage"(floured and deep-fried chicken thigh with skin); [11290] "Karaage"(floured and deep-fried chicken thigh without skin); [11292] chicken nuggets; [11293] "Tsukune" (Japanese chicken meatball); [12017] Tamago-dofu (cold savory egg custard); [12018] Tamago-yaki (Rolled omelet), Atsuyaki-tamago (sweet rolled omelet with Katsuo-bushi and kombu dashi); [12019] Tamago-yaki (Rolled omelet), Dashimaki-tamago (rolled omelet with Katsuo-bushi and kombu dashi)

Table S1. *Continued*

Food Group	Examples (Food codes in brackets are based on the Standard Tables of Food Composition in Japan 2020 –Eighth revision–)
Dairy products	[13007] Milk beverages, coffee flavored; [13015] cream substitute, milk and vegetable fats; [13016] cream substitute, vegetable fat; [13017] whipping cream, milk fat, with added sugar; [13018] whipping cream substitute, milk and vegetable fats, with added sugar; [13019] whipping cream substitute, vegetable fat, with added sugar; [13020] coffee whitener, liquid, milk fat; [13022] coffee whitener, liquid, vegetable fat; [13023] coffee whitener, powder, milk fat; [13024] coffee whitener, powder, vegetable fat; [13040] cheeses, processed; [13042] ice cream, high fat; [13043] ice cream, regular; [13044] ice cream, ice milk; [13045] ice cream, lacto-ice, regular; [13046] ice cream, lacto-ice, low fat; [13047] ice cream, soft-serve; [13049] sherbet
Confectioneries	[1033] Raisin bread; [15005] "Imagawayaki" (Japanese waffle stuffed with red bean paste); [15009] "Kasutera" (rectangle sponge cake); [15023] "Daifuku-mochi" (sweet rice cake stuffed with red bean paste); [15027] "Dorayaki" (Japanese pancake sandwich with red bean paste filling); [15033] "Mushi-manju" (steamed sweet dough stuffed with red bean paste); [15035] Chinese style steamed bun, stuffed with meat and vegetable; [15041] "Amedama" (sugar candy); [15057] "Age-senbei" (fried and salted rice cracker); [15059] "Arare" (glutinous rice cracker); [15060] "Shoyu-senbei" (soy sauce flavored rice cracker); [15069] baked bun with red bean paste filling, regular; [15070] baked bun with custard cream filling, regular; [15073] custard cream puff; [15074] sponge cake; [15075] layered cream cake; [15076] Danish pastry; [15077] doughnuts, yeast-leavened; [15078] doughnuts, cake-type; [15079] pie pastry; [15080] apple pie; [15082] butter cake; [15083] thick pancake; [15086] caramel custard pudding; [15087] orange jelly; [15095] sablé shortbread; [15096] puff pastry biscuits; [15097] hard biscuits; [15098] soft biscuits; [15101] wheat flour snack, extruded; [15102] corn snack, extruded; [15103] potato chips, regular; [15104] potato chips, fabricated; [15105] caramel soft candy; [15106] compressed tablet candy; [15110] sugar candy; [15113] marshmallows; [15114] chocolate-covered biscuit; [15115] white chocolate; [15116] milk chocolate; [15119] Chewing gum, sugar-coated; [15127] fried bun with curry filling; [15132] "Melon-pan" (sweet bun covered in a thin layer of crisp cookie dough); [15134] cheesecake, baked; [15137] chocolate with almonds; [15138] pastry cream; [15141] wafers with cream; [15142] jelly made from konjac

Table S1. *Continued*

Food Group	Examples (Food codes in brackets are based on the Standard Tables of Food Composition in Japan 2020 –Eighth revision–)
Alcoholic beverages	[16001] "Sake", regular; [16002] "Sake", "Junmai" (made with only rice, water and koji) ; [16003] "Sake", "Honjozo" (made with rice, water, koji and distilled alcohol. The rice used must be polished to at least 70%) ; [16006] beer, pale; [16009] "Happoshu" (beer-like beverage with less than 67% malt content); [16010] white wine; [16011] red wine; [16014] "Shochu", distilled through a continuous still; [16015] "Shochu", distilled through a pot still; [16016] whisky; [16023] synthetic "Sake"; [16025] "Mirin" (sweet liquor made from rice, rice koji and Shochu or distilled alcohol), regular; [16059] lemon-flavored canned shochu highball
Non-alcoholic beverages	[7004] Acerola 10 % fruit juice beverage; [16047] ready-to-drink canned coffee with milk and sugar; [16049] cocoa; [16051] "Kobu-cha" (kombu powder for drink); [16052] fruit flavored and colored carbonated beverage; [16053] cola; [16054] clear soda; [16057] sports drink; [16058] beer-flavored carbonated drink, alcohol free
Fats and oils	[14020] Margarine, soft type, home use; [14021] fat spread; [14022] shortening, home use; [14029] margarine, soft type, commercial use; [14030] shortening, commercial use for confectionery; [17039] soy sauce based, fat-free; [17040] French dressing; [17041] thousand island dressing; [17042] mayonnaise, whole egg type; [17043] mayonnaise, egg yolk type; [17116] soy sauce based, with oil; [17117] sesame dressing; [17118] mayonnaise-type dressing, low calorie type
Seasonings and spices	[3026] glucose fructose syrup ; [17001] Japanese Worcester sauce, common type; [17002] Japanese Worcester sauce, semi-thick type; [17003] Japanese Worcester sauce, thick type; [17004] Doubanjiang; [17006] Chinese chili oil; [17007] "Koikuchi-shoyu" (common soy sauce); [17008] "Usukuchi-shoyu" (light color soy sauce); [17009] "Tamari-shoyu" (full-bodied soy sauce); [17011] "Shiro-shoyu" (extra light color soy sauce); [17015] grain vinegar; [17016] rice vinegar; [17018] fruit vinegar, cider vinegar; [17019] "Katsuo-bushi dashi" (stock of "Katsuo-bushi"); [17020] "kombu dashi" (stock of dried kombu); [17021] "Katsuo-bushi and kombu dashi" (stock of "Katsuo-bushi" and dried kombu) ; [17022] "Shiitake dashi" (stock of dried Shiitake mushroom); [17023] "Niboshi dashi" (stock of small dried sardine); [17024] chicken bone stock; [17025] chicken, pork and vegetable stock; [17026] beef and vegetable stock; [17027] meat and vegetable stock cubes; [17028] stock powder, "Katsuo-bushi"; [17029] Japanese noodle soup, non-concentrated (soy sauce base); [17030] Japanese noodle soup, triple-concentrated (soy sauce base); [17031] oyster sauce; [17032] mapo tofu sauce; [17033] meat sauce; [17036] ketchup; [17037] tomato sauce; [17049] instant miso soup, powdered type; [17050] instant miso soup, paste type;

Table S1. *Continued*

Food Group	Examples (Food codes in brackets are based on the Standard Tables of Food Composition in Japan 2020 –Eighth revision–)
Seasonings and spices	[17051] Japanese curry roux, instant; [17052] hash and rice roux, instant; [17053] "Sakekasu" (sake lees); [17054] mirin-like sweet cooking seasoning; [17056] onion powder; [17058] mustard, paste ; [17059] mustard, yellow mustard; [17060] mustard, whole grain mustard; [17076] garlic paste; [17081] wasabi paste ; [17085] Japanese Worcester sauce, sweet thick type for "Okonomiyaki" (Japanese-style savory pancake with various ingredients); [17086] salt-reduced soy sauce; [17087] pre-seasoned soy sauce with soup stock; [17090] black rice vinegar; [17092] stock powder for "Oden" (Japanese winter hodgepodge); [17093] stock powder, chicken, pork and vegetable; [17094] sweet vinegar; [17098] sesame sauce; [17099] "Sanbaizu" (vinegar mixture containing sesami, soy sauce and mirin); [17101] sweetened vinegar for "Inarizushi" (fried tofu pouch filled with sushi rice) ; [17103] sweetened vinegar for "Makizushi" (rolled sushi) and "Hakozushi"(pressed sushi); [17104] Chinese style vinegar; [17105] demi-glace sauce; [17106] Tian Mian Jiang (sweet soybean paste); [17108] seasoning sauce for "Hiyashi-chuka" (ramen noodles in a cold sweet soy sauce broth topped with meat and vegetables) ; [17109] white sauce; [17110] ponzu vinegar with soy sauce; [17112] seasoning sauce for "Yakitori" (grilled chicken skewers); [17113] barbecue sauce, soy sauce based; [17115] "Yuzu kosho" (spicy paste made from chili, yuzu zest and salt); [17119] salt reduced miso; [17120] pre-seasoned miso with soup stock; [17124] "Neri-miso" (miso sauce containing egg and mirin) ; [17125] seasoning mix for "Ochazuke" (bowl of rice soaked in dashi broth); [17126] instant soup mix, "Sumashi-jiru" (Japanese traditional clear-soup); [17127] "Furikake" (Seasoning mix for rice, containing dried seaweed and egg); [17130] ago dashi (stock of dried flying fish); [17136] kimuchi seasoning; [17137] ponzu vinegar with soy sauce, commercial products; [17138] cooking wine
Pickles	[6143] Japanese radishes, Daikon, root, pickles, "Fukujin-zuke" (pickled with Daikon, eggplant, immature sword pods and east Indian lotus rhizome)

[1]Among the 421 food codes of highly processed foods in the 8-day dietary record of 2742 participants, the 227 food codes that appeared ≥100 times are shown.

Table S2. Associations of highly processed food consumption when foods consumed as a dish consisting of multiple ingredients are classified as highly processed food (high-estimate scenario) with basic characteristics of Japanese adults (*n* = 2742)

Variables	% of grams				% of energy			
	Regression coefficient	95% confidence interval		*p*[1]	Regression coefficient	95% confidence interval		*p*[1]
Age group (y)								
18–39	Ref	-	-	-	Ref	-	-	-
40–59	-3.39	-4.54	-2.23	<0.0001	-5.45	-6.64	-4.26	<0.0001
60–79	-10.19	-11.52	-8.86	<0.0001	-13.21	-14.58	-11.85	<0.0001
Sex								
Male	Ref	-	-	-	Ref	-	-	-
Female	-3.03	-4.10	-1.97	<0.0001	-0.24	-1.34	0.86	0.67
BMI (kg/m^2)								
Q1 (median: 19.8)	Ref	-	-	-	Ref	-	-	-
Q2 (median: 22.5)	2.01	0.07	3.95	0.04	2.10	0.10	4.09	0.04
Q3 (median: 26.1)	1.62	-0.50	3.74	0.13	2.83	0.65	5.00	0.01
Annual household income (%)[2]								
<4 million Japanese yen	Ref	-	-	-	Ref	-	-	-
≥4 to <7 million Japanese yen	0.62	-0.51	1.75	0.28	-0.20	-1.36	0.96	0.74
≥7 million Japanese yen	1.36	0.17	2.54	0.03	-0.04	-1.26	1.18	0.95
Educational level (%)								
Junior high school or high school	Ref	-	-	-	Ref	-	-	-
College or technical school	0.94	-0.23	2.11	0.12	1.14	-0.07	2.34	0.06
University or higher	1.03	-0.15	2.22	0.09	1.13	-0.09	2.35	0.07
Smoking status (%)								
Current smoker	Ref	-	-	-	Ref	-	-	-
Past smoker	-2.82	-4.35	-1.30	0.0003	-2.48	-4.05	-0.92	0.002
Never smoker	-5.45	-6.81	-4.09	<0.0001	-5.26	-6.66	-3.86	<0.0001
Employment status (%)								
Unemployed (including students)	Ref	-	-	-	Ref	-	-	-
Part-time job	4.11	2.45	5.77	<0.0001	3.53	1.82	5.23	<0.0001
Full-time job	10.43	9.04	11.81	<0.0001	8.24	6.82	9.66	<0.0001
Physical activity level (MET × h)								
Q1 (median: 33.3)	Ref	-	-	-	Ref	-	-	-
Q2 (median: 38.3)	-0.84	-1.97	0.29	0.15	-1.05	-2.21	0.11	0.08
Q3 (median: 45.0)	-0.52	-1.67	0.62	0.37	-0.12	-1.30	1.06	0.84

MET, metabolic equivalent; SD, standard deviation; T, tertile. [1]Multivariable linear regression was used including all variables in the model simultaneously. [2]The subjects were categorized into tertiles.
